# Establishment of an in vitro culture and regeneration protocol for the native Chilean grass *Polypogon australis* Brong

**DOI:** 10.1038/s41598-026-61154-w

**Published:** 2026-09-04

**Authors:** Javiera Venegas-Rioseco, Rosanna Ginocchio, Claudia Ortiz-Calderón, Götz Hensel

**Affiliations:** 1 Departamento de Ecosistemas y Medio Ambiente, Facultad de Agronomía y Sistemas Naturales, Pontificia Universidad Católica de Chile, Santiago, Chile; 2 Center of Applied Ecology and Sustainability, Pontificia Universidad Católica de Chile, Santiago, Chile; 3 Laboratorio de Bioquímica Vegetal y Fitorremediación, Departamento de Biología, Facultad de Química y Biología, Universidad de Santiago de Chile, Santiago, Chile; 4 Centre for Plant Genome Engineering, Faculty of Mathematics and Natural Sciences, Heinrich Heine Universität, Düsseldorf, Germany; 5 Cluster of Excellence in Plant Sciences, Heinrich Heine University Duesseldorf, Düsseldorf, Germany

**Keywords:** Biological techniques, Biotechnology, Plant sciences

## Abstract

*Polypogon australis* Brong. is a native Chilean grass frequently found colonizing metal-rich mine tailings, yet it lacks an established in vitro regeneration system to support controlled physiological and biotechnological studies. Here, we report a reproducible protocol for seed germination, callus induction, and plant regeneration using coleoptile–mesocotyl explants. Surface-sterilized seeds were germinated on Murashige and Skoog (MS) medium supplemented with sucrose, achieving a cumulative germination percentage of 47.67 ± 3.15% after 15 days. The coleoptile–mesocotyl explant proved highly responsive to culture on callus induction medium (CIM) supplemented with dicamba, resulting in a callus induction frequency of 30.55 ± 11.96% after 3–5 weeks. Induced calli were predominantly embryogenic, with embryogenic calli representing 65.42 ± 8.61% of the total callus population. Embryogenic calli regenerated complete plantlets with a regeneration efficiency of 45.0 ± 23.3%. Organogenic structures, including primary shoots and roots, developed directly from embryogenic calli maintained on callus induction medium (CIM) supplemented with dicamba, without transfer to a specialized regeneration medium containing organogenesis-promoting growth regulators. After the initiation of organogenesis, cultures were exposed to a 16 h light/8 h dark photoperiod while remaining on CIM, and regenerated plantlets were subsequently transferred to MS+10 S medium for further growth and elongation. This study establishes the first complete in vitro regeneration system for *P. australis*, providing a practical framework for future physiological studies, large-scale propagation, genetic transformation, and genome engineering applications in this ecologically relevant native Chilean grass.

## Introduction

Native plant species that colonize metal-enriched soils, including abandoned mine tailings, often display specialized physiological and biochemical adaptations that enable survival under high concentrations of potentially toxic elements^1,2^. These facultative metallophytes hold considerable promise for phytotechnologies such as phytostabilization, phytoextraction, and phytomining, owing to their ability to accumulate, tolerate, or immobilize metals in a cost-effective and environmentally sustainable manner^3^. In addition to their applied relevance, these species serve as informative models for studying metal homeostasis and stress tolerance under extreme environmental conditions.

The development of efficient and reproducible in vitro propagation systems is fundamental for advancing both basic and applied research in metallophytes. Tissue culture methodologies enable large-scale clonal propagation, provide homogeneous plant material for physiological and biochemical studies, and serve as platforms for downstream molecular and functional analyses^4^. However, many native grasses exhibit strong recalcitrance to in vitro culture, making the establishment of regeneration systems particularly challenging^5,6^. Despite these limitations, recent advances demonstrate that somatic embryogenesis can be achieved in several non-model Poaceae species when explant selection, auxin balance, and micronutrient availability are optimized. For instance, *Cenchrus ciliaris* now possesses robust embryogenic callus and regeneration systems derived from juvenile tissues^7^, while *Chloris gayana* has recently been regenerated and transformed using optimized *Agrobacterium*-mediated protocols^8^. In addition, long-term embryogenic competence has been achieved in *Miscanthus × giganteus* through fine-tuning of micronutrients such as CuSO_4_^9,10^. These studies collectively highlight the feasibility of overcoming recalcitrance in diverse Poaceae lineages.

Among native grasses adapted to extreme environments, *Polypogon australis* Brong. is a native, non-endemic species widely distributed across Chile and other regions of South America^11^. The species frequently colonizes disturbed environments, including copper-rich mine tailings, riverbanks, and marginal soils, where it demonstrates tolerance to multiple abiotic stressors^12,13^. Under controlled conditions, *P. australis* has been shown to accumulate metals such as copper and zinc and to exhibit enhanced antioxidant responses when exposed to metal-enriched substrates^14^. These characteristics highlight its role as a facultative metallophyte and support its potential for phytoremediation applications.

Despite its ecological relevance and phytotechnological potential, *Polypogon australis* remains underrepresented in plant biotechnology due to the absence of a validated in vitro regeneration system. The lack of reliable protocols for callus induction and plant regeneration has limited its use in controlled physiological studies, large-scale propagation, and future genetic transformation approaches. Importantly, regeneration and transformation capabilities have recently been demonstrated within the genus. Zhou et al.^15^ established callus induction and *Agrobacterium*-mediated transformation in *Polypogon fugax*, indicating that members of the genus possess developmental competence suitable for tissue culture-based biotechnology. Likewise, successful regeneration has been reported in other native South American grasses, including *Spartina argentinensis*^16^, supporting the broader feasibility of developing regeneration systems in underrepresented Poaceae species adapted to extreme environments.

Given the ecological importance of *P. australis* and its emerging relevance for phytoremediation and functional genomics, the development of a reproducible regeneration platform represents a critical step toward its incorporation into biotechnological research. Therefore, the objective of this study was to establish a complete in vitro culture and regeneration system for *P. australis* by optimizing seed germination, identifying a responsive coleoptile–mesocotyl explant source for embryogenic callus induction, and regenerating complete plantlets capable of normal shoot and root development. This regeneration system provides a foundation for future studies on copper tolerance, phytoremediation, and genome engineering in this native metallophyte.

## Results

### Seed germination and early seedling development

**Fig. 1:**
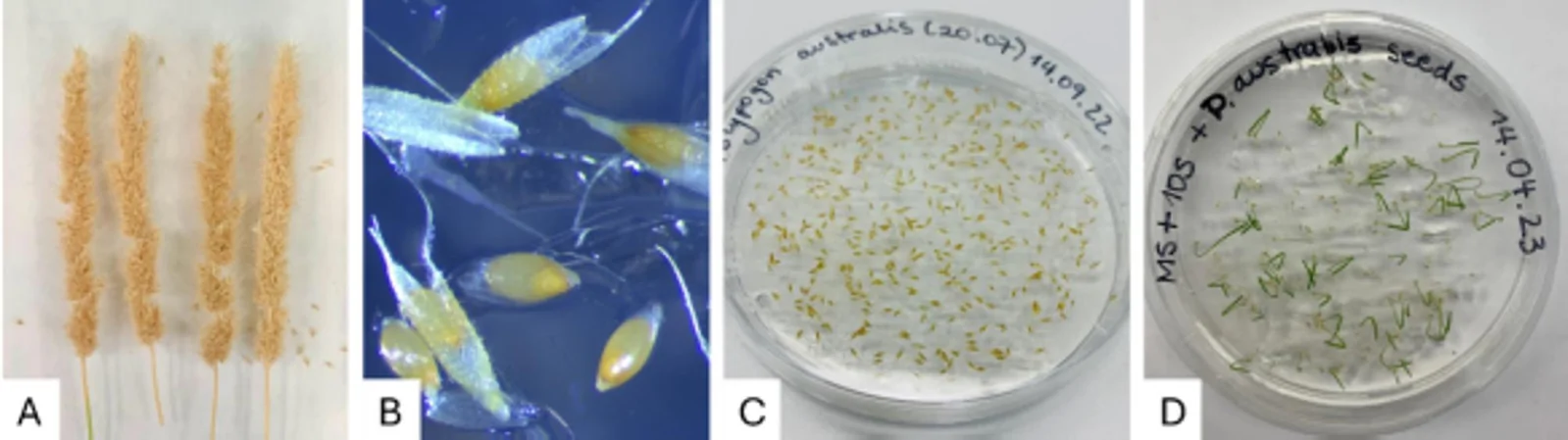
Plant material, seed germination, and early seedling development of *Polypogon australis* under in vitro culture conditions. (**A**) Mature spikes of *P. australis* used as the source of seeds for in vitro culture establishment. (**B**) Seeds of *P. australis* used for in vitro culture establishment. (**C**) Surface-sterilized seeds cultured on Murashige and Skoog (MS) medium during germination. (**D**) Early seedling growth under in vitro culture conditions following germination. These stages provided the plant material used for subsequent explant excision and callus induction experiments.

Surface-sterilized seeds of *Polypogon australis* germinated successfully on Murashige and Skoog (MS) medium supplemented with sucrose and MS vitamins (Figs. 1 and 3A). Germination was first observed between 5 and 7 days after sowing (DAS), and cumulative germination increased significantly throughout the evaluation period. Mean germination percentages reached 21.1 ± 2.06% at 8 DAS, 30.9 ± 2.88% at 11 DAS, and 47.67 ± 3.15% at 15 DAS, indicating that the culture conditions supported reliable seed germination and seedling establishment. Significant differences in cumulative germination percentage were detected among evaluation dates (Tukey’s test, *p* < 0.05), reflecting the progressive establishment of seedlings during the first 15 days of culture.

**Fig. 2:**
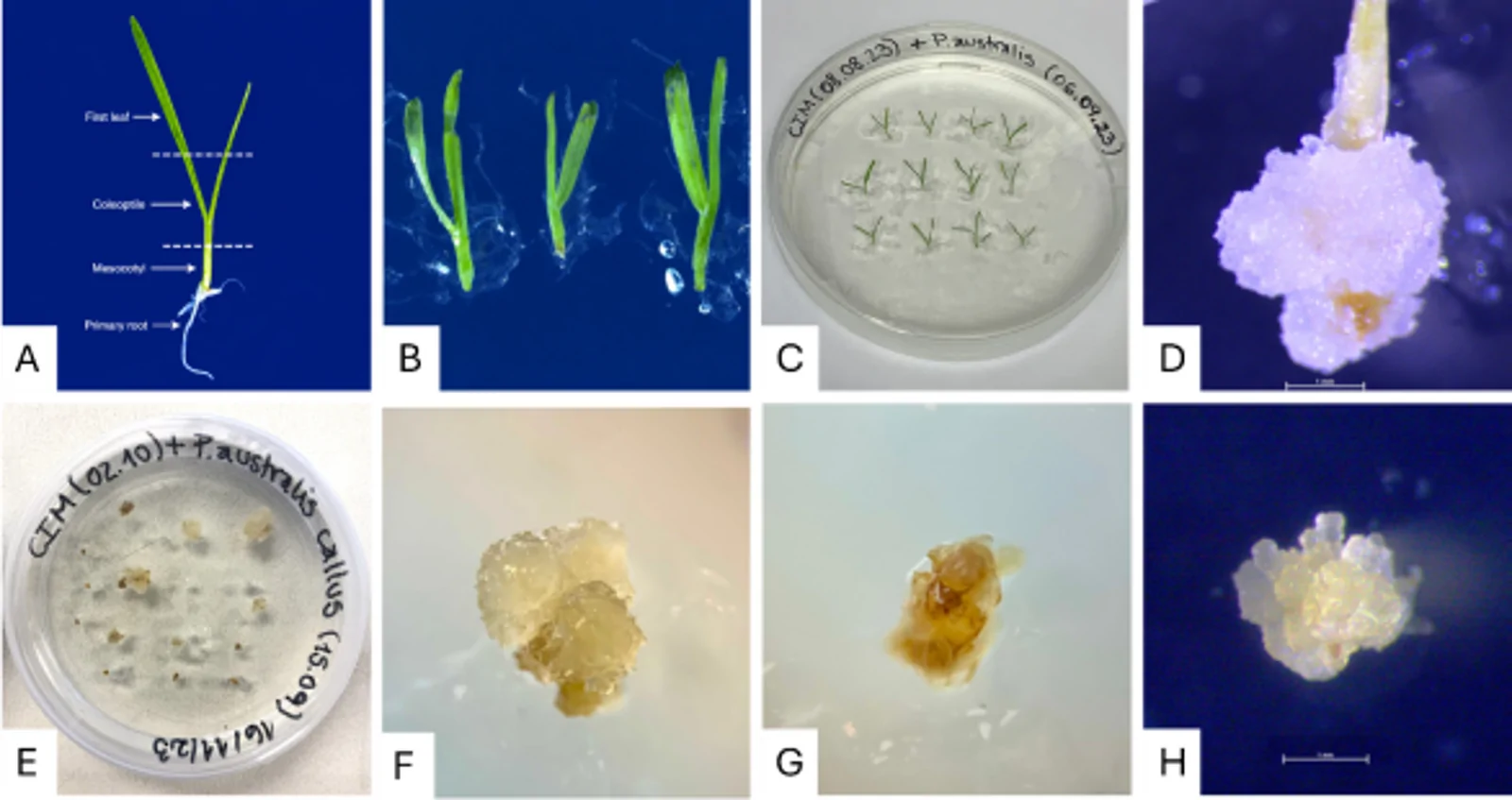
Morphological characterization of seedling-derived explants and callus induction in *Polypogon australis*. (**A**) Morphological diagram of a 7–10-day-old seedling indicating the first leaf, coleoptile, mesocotyl, and primary root. Dashed lines indicate the region used for explant excision. (**B**) Representative coleoptile–mesocotyl explants excised from in vitro-grown seedlings prior to culture. (**C**) Explants cultured on callus induction medium (CIM) for embryogenic callus induction. (**D**) Early embryogenic callus formation at the basal region of the explant following culture on CIM. (**E**) Representative culture plate showing callus induction from coleoptile–mesocotyl explants after 3–5 weeks of culture on CIM. (**F**) Representative embryogenic callus displaying a compact, nodular, and translucent morphology. (**G**) Representative non-embryogenic callus exhibiting a soft, irregular, and disorganized morphology. (**H**) Mature embryogenic callus characterized by a compact structure and granular surface morphology. Scale bars = 1 mm (**D**, **H**).

**Fig. 3:**
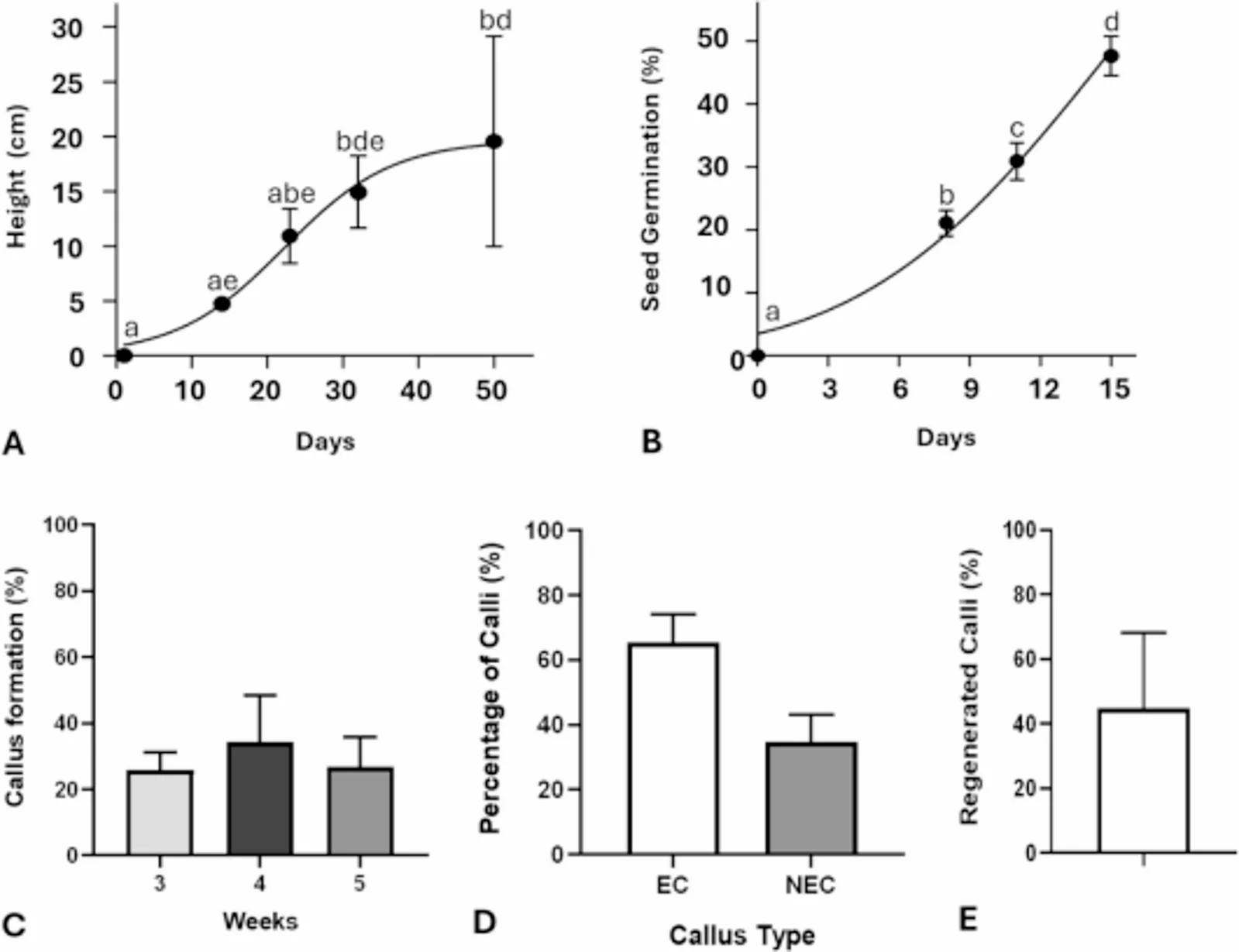
Quantitative evaluation of germination, growth, callus induction, callus type distribution, and regeneration in *Polypogon australis*. (**A**) Cumulative germination percentage of seeds cultured under in vitro conditions over 15 days after sowing (DAS). (**B**) Mean seedling height during early in vitro growth, reaching 19.55 ± 9.58 cm at 50 DAS. (**C**) Callus induction frequency (%) obtained from coleoptile–mesocotyl explants after 3–5 weeks of culture on callus induction medium (CIM), with a mean induction frequency of 30.55 ± 11.96%. (**D**) Distribution of embryogenic (EC) and non-embryogenic calli (NEC) within the induced callus population, with EC representing 65.42 ± 8.61% and NEC 34.58 ± 8.61% of total calli (*n* = 3). (**E**) Regeneration efficiency of embryogenic calli, expressed as the percentage of embryogenic calli producing shoots and roots relative to the total number of embryogenic calli evaluated, with a mean regeneration efficiency of 45.0 ± 23.3% (*n* = 7).

Following germination, seedlings exhibited normal morphological development characterized by elongation of the coleoptile, emergence of the first leaf, and development of the primary root system (Figs. 1D and 2A). Seedlings transferred to MS+10 S medium maintained continuous growth throughout the 50-day evaluation period. Mean seedling height reached 19.55 ± 9.58 cm at 50 DAS (Fig. 3B), providing sufficient biomass and uniform plant material for subsequent explant excision and tissue culture experiments.

### Callus induction from coleoptile–mesocotyl explants

The coleoptile–mesocotyl region of 7–10-day-old seedlings was identified as the most responsive explant source for callus induction (Fig. 2). Following excision and culture on callus induction medium (CIM) supplemented with 2.5 mg L⁻¹ dicamba, early callus formation was observed at the basal region of the explants after approximately 12 days of incubation in darkness (Fig. 2D).

Callus proliferation continued during the subsequent weeks of culture, producing visible callus masses after 3–5 weeks on CIM (Fig. 2E). The mean callus induction frequency was 30.55 ± 11.96% (Fig. 3C). Calli ranged from approximately 1 to 3 mm in diameter and displayed distinct morphological characteristics that allowed classification into embryogenic and non-embryogenic types.

Embryogenic calli (EC) exhibited a compact, nodular, and translucent appearance (Fig. 2F and H), whereas non-embryogenic calli (NEC) displayed a softer, irregular, and less organized morphology (Fig. 2G). Classification of induced calli showed that embryogenic calli represented 65.42 ± 8.61% of the total callus population, whereas non-embryogenic calli accounted for 34.58 ± 8.61% (*n* = 3) (Fig. 3D). The predominance of embryogenic calli demonstrated the suitability of the coleoptile–mesocotyl explant and the CIM composition for inducing morphogenic tissues in *P. australis*.

### Regeneration of embryogenic calli

**Fig. 4:**
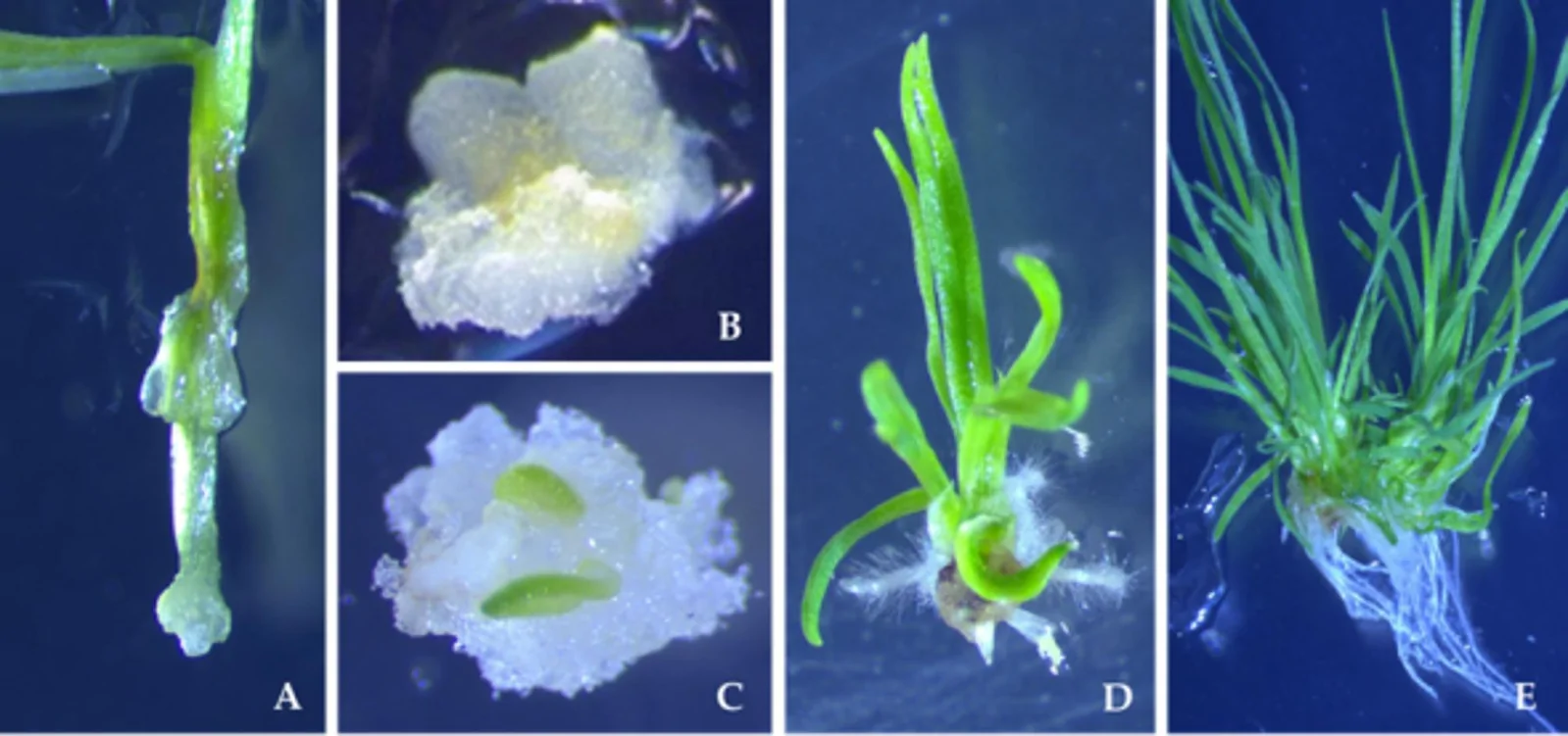
Regeneration sequence of embryogenic calli in *Polypogon australis*. (**A**) Embryogenic callus developing at the basal region of the coleoptile–mesocotyl explant. (**B**) Proliferating embryogenic callus following culture on callus induction medium (CIM). (**C**) Emergence of green regenerative structures from embryogenic calli. (**D**) Shoot development during in vitro regeneration. (**E**) Regenerated plantlet exhibiting multiple shoots and a well-developed root system following transfer to MS+10 S medium for shoot and root elongation.

Embryogenic calli were capable of regenerating complete plantlets under the culture conditions employed. The first organogenic structures, including primary shoots and roots, emerged directly from embryogenic calli maintained on CIM supplemented with dicamba under darkness (Fig. 4C). Following the initiation of organogenesis, cultures were transferred to a 16 h light/8 h dark photoperiod while remaining on CIM, which supported continued shoot and root development (Fig. 4D).

Regeneration efficiency, calculated as the percentage of embryogenic calli producing shoots and roots relative to the total number of embryogenic calli evaluated, reached 45.0 ± 23.3% (*n* = 7) (Fig. 3E). Individual embryogenic calli frequently produced multiple shoots, demonstrating the regenerative competence of the induced tissues.

Once regenerated structures were established, regenerating calli were transferred to MS+10 S medium to promote further plantlet growth and elongation.

Regenerated plantlets displayed normal morphological development, including elongation of leaves and formation of a functional root system (Fig. 4E). Regeneration efficiency, calculated as the percentage of embryogenic calli producing shoots and roots, reached 45.0 ± 23.3% (*n* = 7) (Fig. 3E). Individual embryogenic calli frequently generated multiple shoots, demonstrating the regenerative competence and totipotency of the induced tissues.

Following transfer of regenerating calli to MS+10 S medium under a 16 h light/8 h dark photoperiod, regenerated plantlets continued shoot and root elongation, reaching approximately 5–7 cm in height and developing a functional root system (Fig. 4E). Together, these results establish a reproducible regeneration system for *P. australis*, encompassing seed germination, embryogenic callus induction, and complete plant regeneration under controlled in vitro conditions. Together, these results establish a reproducible regeneration system for *P. australis*, encompassing seed germination, embryogenic callus induction, and complete plant regeneration under controlled in vitro conditions.

## Discussion

This study establishes the first reproducible in vitro regeneration system for *Polypogon australis*, a native metallophyte frequently associated with copper-enriched environments^12,13^. By optimizing seed germination, explant selection, and auxin-mediated callus induction, we demonstrated that *P. australis* possesses a reliable regenerative capacity under controlled culture conditions. The successful induction of embryogenic calli and subsequent regeneration of complete plantlets provides an essential methodological foundation for future physiological, molecular, and biotechnological studies in this species.

Efficient seed germination and early seedling development are critical prerequisites for tissue culture systems because they provide uniform source material for explant preparation^4^. Under the culture conditions employed, cumulative germination reached 47.67 ± 3.15% after 15 days, while seedlings exhibited normal morphological development and vigorous growth. The successful establishment of seedlings under sterile conditions ensured a consistent source of coleoptile–mesocotyl explants and minimized variation associated with heterogeneous field-derived material. Similar germination-based approaches have been successfully employed in other Poaceae species, where uniform seedling establishment represents a key factor influencing subsequent callus induction and regeneration efficiency^17^.

The coleoptile–mesocotyl explant proved highly responsive for callus induction and represented the most suitable tissue source for subsequent regeneration. Juvenile tissues frequently display elevated morphogenic competence due to their active cellular division and developmental plasticity, characteristics that have been exploited in numerous grass tissue culture systems^4,6^. Similar responses have been reported in species such as *Cenchrus ciliaris*^7^, *Chloris gayana*^8^, and *Miscanthus × giganteus*^9,10^, where successful regeneration depends strongly on the use of young, actively growing tissues. The use of dicamba as the sole growth regulator promoted callus formation and supported the development of embryogenic tissues, consistent with its widespread application in Poaceae tissue culture protocols^6,7^. The observed callus induction frequency of 30.55 ± 11.96% demonstrates that *P. australis* can successfully undergo dedifferentiation and embryogenic transition under relatively simple culture conditions.

A notable feature of the protocol was the predominance of embryogenic calli among the induced callus population. Embryogenic calli accounted for 65.42 ± 8.61% of all induced calli and displayed compact, nodular, and translucent morphologies characteristic of morphogenic tissues. In contrast, non-embryogenic calli represented only 34.58 ± 8.61% of induced calli and exhibited irregular and less organized growth patterns. The predominance of embryogenic tissues is particularly relevant because regeneration efficiency in grasses is frequently constrained by the proportion of calli capable of maintaining developmental competence during prolonged culture^6,7^. The high proportion of embryogenic calli obtained in this study suggests that the selected explant type and culture conditions effectively promoted morphogenic development.

Following embryogenic callus induction, embryogenic calli successfully regenerated complete plantlets with normal shoot and root development. Regeneration efficiency reached 45.0 ± 23.3%, and individual calli frequently produced multiple shoots. Notably, the first organogenic structures, including shoots and roots, emerged directly from embryogenic calli maintained on callus induction medium supplemented with dicamba under darkness, without transfer to a specialized regeneration medium containing organogenesis-promoting growth regulators. Following the initiation of organogenesis, cultures were exposed to a 16 h light/8 h dark photoperiod while remaining on CIM, allowing continued shoot and root development before transfer to MS+10 S medium for plantlet elongation. These observations suggest that the developmental program initiated during callus induction was sufficient to support subsequent organogenesis and plant formation. Similar regeneration patterns have been reported in other Poaceae species and are generally considered indicators of a stable and reproducible regeneration system^7,8,16^. Furthermore, the development of complete plantlets from embryogenic tissues confirms the maintenance of cellular totipotency throughout the culture process.

The establishment of a reliable regeneration platform for *P. australis* is particularly significant given the ecological characteristics of this species. As a facultative metallophyte capable of colonizing copper-rich mine tailings^12,13^, *P. australis* represents a promising model for investigating metal tolerance, metal accumulation, and oxidative stress responses under controlled experimental conditions. Previous studies have demonstrated the capacity of *P. australis* to accumulate metals and activate antioxidant defense mechanisms under metal stress^14^, highlighting its relevance for phytoremediation research. In vitro culture systems provide genetically and physiologically uniform material that facilitates the study of these processes while reducing environmental variability associated with field-grown plants^4^. Consequently, the protocol described here expands the experimental tools available for investigating the mechanisms underlying metal tolerance in native metallophytes.

Beyond its value for physiological studies, the generation of embryogenic calli provides the tissue foundation required for genetic transformation and genome engineering approaches. Regeneration capacity remains one of the principal limitations affecting transformation efficiency in many grass species^6,7^. Therefore, the successful regeneration of *P. australis* plantlets represents a critical step toward the development of *Agrobacterium*-mediated transformation systems and future CRISPR-based applications targeting genes involved in metal homeostasis, stress tolerance, and phytoremediation. The recent demonstration of callus induction and *Agrobacterium*-mediated transformation in *Polypogon fugax* further supports the feasibility of implementing similar approaches in *P. australis*^15^.

In conclusion, this study provides the first complete regeneration system for *Polypogon australis*, encompassing seed germination, embryogenic callus induction, and plant regeneration under controlled in vitro conditions. The protocol establishes a practical framework for future physiological studies, large-scale propagation, genetic transformation, and genome engineering applications in this native Chilean grass. By overcoming a key methodological limitation, this work expands the biotechnological potential of *P. australis* and supports its future use in phytoremediation research and functional genomics.

## Methods

### Plant material and seed sterilization

The plant material used in this study originated from seeds obtained from an experimental nursery. These nursery seeds were originally sourced from wild populations naturally growing on a copper mine tailings storage facility located at Planta Manuel Antonio Matta, Atacama Region, northern Chile (27.3° S, 70.3° W)^12^. No wild plants were collected specifically for this study. All plant material was obtained and used in accordance with relevant institutional, national, and international guidelines and regulations. Seeds from the nursery population were used as the starting material for all in vitro experiments.

Seed surface sterilization was performed following the protocol described by Acemi and Özen^18^, with modifications to sodium hypochlorite concentration and exposure time. Seeds were immersed in 70% (v/v) ethanol for 1 min, followed by 1% (w/v) sodium hypochlorite supplemented with one drop of Tween™ 20 for 10 min. Subsequently, seeds were rinsed three times with sterile distilled water under aseptic conditions.

### Seed germination and early seedling growth

Surface-sterilized seeds were cultured on Murashige and Skoog (MS) basal medium^19^ supplemented with 1% (w/v) sucrose, MS vitamins, and 0.43% (w/v) Phytagel^®^ (MS+10 S). Approximately 400–500 mg of seeds were sterilized per batch, and four biological replicates containing an average of 190 seeds per Petri dish were established for the germination assay. Following sowing, plates were incubated in darkness at 4 °C for 24 h and subsequently transferred to a growth chamber maintained at 24 °C under a 16 h light/8 h dark photoperiod with a light intensity of 136 µmol m⁻² s⁻¹. The pH of the medium was adjusted to 5.75–5.8 prior to autoclaving.

Germination dynamics were evaluated following the approach described by Noni-Morales et al.^17^. Cumulative germination percentage was recorded at 8, 11, and 15 days after sowing (DAS) and calculated as the number of germinated seeds relative to the total number of seeds sown per plate, expressed as a percentage.

For early growth assessments, germinated seedlings were transferred to 1-L culture jars containing fresh MS+10 S medium. Five seedlings were cultured per jar, and growth was monitored for 50 days. Three independent biological replicates were established. Seedling height was recorded at the end of the evaluation period.

### Callus induction and regeneration efficiency

Coleoptile–mesocotyl explants were excised from 7–10-day-old seedlings after emergence of the first leaf. Approximately 0.5 cm segments containing the coleoptile–mesocotyl junction were isolated, and roots were removed prior to culture.

Explants were cultured on callus induction medium (CIM) consisting of MS basal salts supplemented with 3% (w/v) maltose, 2.5 mg L⁻¹ dicamba (11.3 µM), 0.10% (w/v) casein hydrolysate, 0.069% (w/v) L-proline, 0.025% (w/v) myo-inositol, and 0.0001% (w/v) thiamine-HCl. The medium was solidified with 0.43% (w/v) Phytagel^®^, and the pH was adjusted to 5.75–5.8 prior to autoclaving.

Cultures were incubated at 24 °C in complete darkness. Early callus formation was assessed after approximately 12 days of culture. Explants were maintained on CIM for 3–5 weeks, with periodic subculture onto fresh medium when required. Callus induction frequency was calculated as the percentage of explants producing callus relative to the total number of cultured explants.

Calli were subsequently classified according to their morphology as embryogenic calli (EC) or non-embryogenic calli (NEC). Embryogenic calli were identified by their compact, nodular, and translucent appearance, whereas non-embryogenic calli exhibited irregular, soft, and less organized morphologies.

### Regeneration of embryogenic calli

Embryogenic calli were capable of regenerating complete plantlets under the culture conditions employed. The first organogenic structures, including primary shoots and roots, emerged directly from embryogenic calli maintained on CIM supplemented with dicamba under darkness (Fig. 4C). Following the initiation of organogenesis, cultures were transferred to a 16 h light/8 h dark photoperiod while remaining on CIM, which supported continued shoot and root development (Fig. 4D).

Regeneration efficiency, calculated as the percentage of embryogenic calli producing shoots and roots relative to the total number of embryogenic calli evaluated, reached 45.0 ± 23.3% (*n* = 7) (Fig. 3E). Individual embryogenic calli frequently produced multiple shoots, demonstrating the regenerative competence and totipotency of the induced tissues.

Once regenerated structures were established, regenerating calli were transferred to MS+10 S medium to promote further plantlet growth and elongation. Following transfer to MS+10 S medium under a 16 h light/8 h dark photoperiod, regenerated plantlets continued shoot and root elongation, reaching approximately 5–7 cm in height and developing a functional root system (Fig. 4E). Together, these results establish a reproducible regeneration system for *P. australis*, encompassing seed germination, embryogenic callus induction, and complete plant regeneration under controlled in vitro conditions.

### Statistical analysis

Statistical analyses were performed using GraphPad Prism version 8.0.1 (GraphPad Software, San Diego, CA, USA). Germination percentages were analyzed using a cumulative Gaussian (inverse normal) model to characterize germination dynamics over time.

For germination assays, four independent biological replicates were evaluated. Early growth experiments consisted of three biological replicates containing five seedlings each. Callus induction assays were performed using five independent culture plates containing 12 explants per plate. Regeneration efficiency was calculated from seven independent embryogenic calli evaluated for shoot and root formation during the regeneration process.

Data are presented as mean ± standard deviation (SD). Differences among treatments or evaluation times were assessed using one-way analysis of variance (ANOVA) followed by Tukey’s multiple comparison test. Statistical significance was established at *p* < 0.05.

## Data Availability

All data generated or analyzed during this study are included in this published article and its supplementary information files. Additional raw data are available from the corresponding author upon reasonable request.Competing interests: The authors declare no competing interests.
